# Effects of diaphragmatic control on the assessment of sniff nasal inspiratory pressure and maximum relaxation rate

**DOI:** 10.1590/bjpt-rbf.2014.0101

**Published:** 2015-11-17

**Authors:** Kadja Benício, Fernando A. L. Dias, Lucien P. Gualdi, Andrea Aliverti, Vanessa R. Resqueti, Guilherme A. F. Fregonezi

**Affiliations:** 1 Laboratório de Desempenho PneumoCardioVascular e Músculos Respiratórios, Departamento de Fisioterapia, Universidade Federal do Rio Grande do Norte (UFRN), Natal, RN, Brazil; 2 Departamento de Fisiologia, Universidade Federal do Paraná (UFPR), Curitiba, PR, Brazil; 3 PneumoCardioVascular Lab, Hospital Universitário Onofre Lopes, Empresa Brasileira de Serviços Hospitalares (EBSERH), UFRN, Natal, RN, Brazil; 4 Dipartimento di Elettronica, Informazione e Bioingegneria, Politecnico Di Milano, Milano, Italy

**Keywords:** respiratory muscles, nasal inspiratory pressure, physical therapy

## Abstract

**OBJECTIVE:**

To assess the influence of diaphragmatic activation control (diaphC) on Sniff Nasal-Inspiratory Pressure (SNIP) and Maximum Relaxation Rate of inspiratory muscles (MRR) in healthy subjects.

**METHOD:**

Twenty subjects (9 male; age: 23 (SD=2.9) years; BMI: 23.8 (SD=3) kg/m^2^; FEV_1_/FVC: 0.9 (SD=0.1)] performed 5 sniff maneuvers in two different moments: with or without instruction on diaphC. Before the first maneuver, a brief explanation was given to the subjects on how to perform the sniff test. For sniff test with diaphC, subjects were instructed to perform intense diaphragm activation. The best SNIP and MRR values were used for analysis. MRR was calculated as the ratio of first derivative of pressure over time (dP/dt_max_) and were normalized by dividing it by peak pressure (SNIP) from the same maneuver.

**RESULTS:**

SNIP values were significantly different in maneuvers with and without diaphC [without diaphC: -100 (SD=27.1) cmH_2_O/ with diaphC: -72.8 (SD=22.3) cmH_2_O; p<0.0001], normalized MRR values were not statistically different [without diaphC: -9.7 (SD=2.6); with diaphC: -8.9 (SD=1.5); p=0.19]. Without diaphC, 40% of the sample did not reach the appropriate sniff criteria found in the literature.

**CONCLUSION:**

Diaphragmatic control performed during SNIP test influences obtained inspiratory pressure, being lower when diaphC is performed. However, there was no influence on normalized MRR.

## Introduction

The evaluation of respiratory muscle strength is an important method for early detection of weakness in these muscles. This evaluation also aims to monitor their function in respiratory, cardiac, and neuromuscular diseases^1^^,^ 2 and provides prognostic and predictive information on survival in different patients^1^^,^ 2. Respiratory muscle strength is estimated using Maximal Expiratory Pressure (MEP) and Maximal Inspiratory Pressure (MIP), which are obtained noninvasively through the mouth and sustained for 2 to 3 seconds with an occluded airway^3^^,^ 4. Despite its usefulness as a diagnostic test, this assessment is difficult for patients with neuromuscular disease, since it requires coordination, collaboration, and facial muscle integrity^4^^,^ 5.

A test has been developed recently to assess inspiratory muscle strength during a sniff (Sniff Nasal Inspiratory Pressure - SNIP). Given that this new method is a natural maneuver performed primarily by the diaphragm in a ballistic as opposed to isometric contraction, it is easily executed when compared to MIP, a maximal sustained static effort. Previous studies consider SNIP a complementary maneuver to MIP because it is a simpler technique that does not require a mouthpiece, since pressure is measured via the nasal airway with a nose clip, making it easier to assess children and patients with neuromuscular disorders^3^^,^ 4.

SNIP has gained clinical importance in recent years, with reference values published for different populations (adults and children)^5^^-^ 8. Studies suggest intense activation of the diaphragm muscle during a maximal sniff^9^^,^ 10. The diaphragm is one of the main inspiratory muscles active during this maneuver, which raises the question of whether to emphasize its action when measuring SNIP. Although the SNIP test is noninvasive, research indicates a high correlation (r=0.99, p<0.001) between this maneuver and invasive techniques measuring esophageal pressure with an esophageal balloon catheter^11^^-^ 13. In addition to assessing muscular strength, SNIP has been used as a predictor of respiratory muscle fatigue by analyzing the Maximum Relaxation Rate (MRR) of inspiratory muscles, calculated based on test kinetics^13^. Previous studies evaluated the MRR in healthy subjects and patients with neuromuscular disorders and chronic obstructive pulmonary disease (COPD)^11^^-^ 13.

The SNIP test is considered a predictor of mortality in patients with COPD and is compared with predictors obtained in more complex assessments related to lung hyperinflation, such as the IC/TLC ratio (Inspiratory Capacity/Total Lung Capacity)^14^. There are a number of studies on SNIP, its clinical importance in cardiorespiratory physical therapy assessment, and methodological description in important guides for respiratory diseases published by scientific institutions^15^^-^ 17. However, there is no information on the need (or not) to stimulate diaphragm contraction by visible abdominal movement. Therefore, the precise technical procedure for the maneuver remains unclear. The aim of this study was to assess the influence of diaphragmatic control (DiaphCtrl) on SNIP and MRR in healthy subjects.

## Method

### Subjects

Twenty healthy subjects aged between 18 and 30 years of both sexes were recruited. Inclusion criteria were: no history of smoking; any neuromuscular, cardiovascular, or respiratory disease that might result in lung dysfunction with spirometric changes; influenza and/or a cold in the week preceding assessment; no regular use of medication to treat respiratory allergies, central nervous system (CNS) depressants, barbiturates, or muscle relaxants; not pregnant; and exhibiting spirometric variables of forced vital capacity (FVC) higher than 80% and the ratio of forced expiratory volume in one second to forced vital volume (FEV_1_/FVC) greater than 85% of the predicted value^14^. Individuals unable to understand and/or correctly perform the required maneuvers or diagnosed with a deviated septum were excluded. All subjects gave their written informed consent in accordance with Resolution 466/12 of the Brazilian National Health Council. The Research Ethics Committee (CEP) of Hospital Universitário Onofre Lopes, Universidade Federal do Rio Grande do Norte (HUOL/UFRN), Natal, RN, Brazil, approved the study under protocol number 185/10.

### Study design

**Figure 1.**
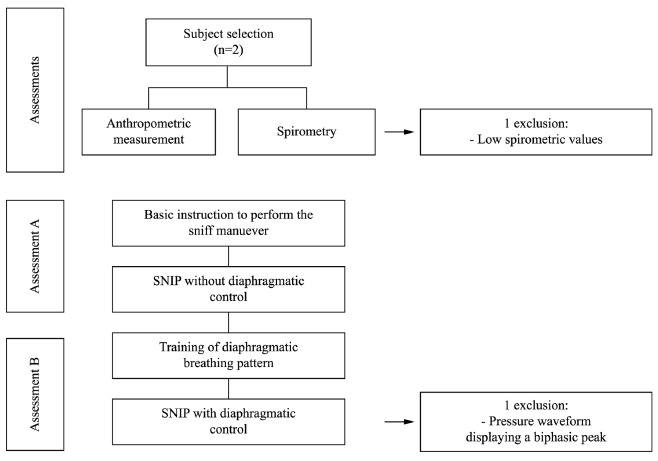
Study design.

This is a cross-sectional, quasi-experimental study. Subjects were submitted to outpatient assessment at the PneumoCardioVascular Performance Laboratory and the PneumoCardioVascular Lab/HUOL/EBSERH, UFRN. After selection, individuals were assessed on the same day for collection of anthropometric and spirometric data to determine their eligibility. SNIP tests were conducted after a 20-minute rest, with a minimum 60-minute interval between the two assessments, followed by MIP measurement. SNIP assessment was conducted twice. On both occasions, the examiner carefully demonstrated the maneuver and then asked the subject to repeat it for familiarization purposes^18^^,^ 19. In assessment A, subjects received only the basic instructions recommended by the American Thoracic Society/European Respiratory Society (ATS/ERS)^16^, which suggests that the sniff maneuver requires little explanation and practice. Subjects executed 5 sniff maneuvers without activating the diaphragm muscle. They were instructed to sniff with maximum effort, followed by a slow, sustained expiration without holding their breath. In assessment B, individuals were trained to breathe in a slow diaphragmatic breathing pattern. They were asked to breathe deeply through their nose, while simultaneously moving the abdominal wall outwards. A period of 5 to 10 minutes was established for training to ensure patients could correctly execute the maneuver. Success was evaluated visually, with maneuvers considered satisfactory when the abdomen clearly expanded on inspiration^20^. After being trained in diaphragmatic breathing, subjects were asked to perform ballistic stomach movements to familiarize themselves with the speed required during the sniff. Next, participants were instructed to perform five consecutive sniffs concomitant to abdominal motion (DiaphCtrl) following the same instructions applied in assessment A (rapid maximum effort, followed by slow and sustained expiration), but emphasizing diaphragm control during execution. In both assessments, the subjects were prompted by being asked to take a "hard sniff". MIP was measured at the end of the test after a 30-minute rest to prevent the static effort required from interfering in obtaining SNIP values. The sequence of measurements was not randomized because, once a maneuver has been taught, it is impossible to ask individuals to execute it without applying the pattern learned and be certain they are performing it as they would have done before training, which could hamper result interpretation. The flow chart is shown in Figure 1.

### Lung function assessment

### Spirometry

Spirometry was performed using a DATOSPIR 120C spirometer (Sibelmed, Barcelona, Spain). Acceptability and reproducibility criteria followed the recommendations of the ATS/ERS^21^ and the guidelines of the Brazilian Pulmonology and Thoracic Society (SBPT)^15^. Assessment was considered complete when three acceptable curves were produced, of which the best two are reproducible (with variation equal to or lower than 5% to 200 ml). The following variables were evaluated during spirometry: forced vital capacity (FVC), and forced expiratory volume in the first second (FEV_1_) and the FEV_1_/FVC ratio. The results were compared to reference values for the Brazilian population^22^.

### Inspiratory muscle strength (MIP and SNIP)

A MicroRPM digital manometer (MICRO medical, Rochester, Kent, United Kingdom) was used to measure the inspiratory pressures MIP and SNIP. Before the start of each test, individuals were instructed on the maneuver, which was then demonstrated by the examiner. The results obtained were compared to reference values for the Brazilian population^22^. Technical criteria of acceptability and reproducibility followed the standards and guidelines of the Brazilian Pulmonology and Thoracic Society (SBPT)^15^.

MIP was measured while participants were seated, with their heads in a neutral position and wearing a nose clip. A disposable cylindrical mouthpiece was coupled to the manometer and positioned firmly between their lips to prevent leakage. Participants were instructed to execute a maneuver for training purposes^19^, and the evaluation was considered complete when three acceptable maneuvers were performed, of which two were reproducible (with variation equal to or lower than 10% of the highest value). A one minute rest was allowed between tests and the highest value of the two reproducible measures obtained was considered for analysis. MIP measurement was based on residual volume (RV), with subjects performing a maximum inspiration.

SNIP assessment was conducted with one nostril occluded by a nose clip, selected according to the size of the subject's nostril and connected to the manometer via a catheter measuring approximately 1 mm. The maneuver was performed from Functional Residual Capacity (FRC), whereby participants executed a maximum sniff through the contralateral (unobstructed) nostril at the end of a slow and sustained expiration^5^. The SNIP test was performed with subjects seated upright, their backs against a chair, knees and hips flexed to 90° and their heads in a neutral position.

Testing was considered complete when 5 acceptable maneuvers had been performed in each assessment (A and B) with a 30-second interval between them^5^. The three best curves for each individual in each assessment were plotted. The test with the highest value was used for statistical analysis, provided it met the criteria described in the literature as suitable for data quantification, namely: peak pressure sustained for less than 50 ms; total sniff duration (T_total_) less than 500 ms; gentle, descending, exponential curve with no biphasic peak^11^.

### Statistical analysis

**Figure 2.**
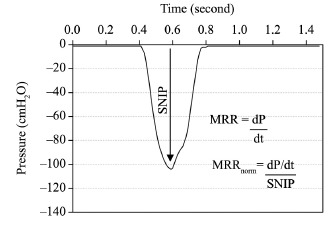
Example of measurement and calculation of Maximum Relaxation Rate of inspiratory muscles (MRR) from Sniff Nasal Inspiratory Pressure (SNIP).

The three best tests from each individual with the highest absolute SNIP value in each assessment (A and B) were chosen for analysis using the LabChart 7 Pro software program (ADInstruments 2009). MRRs were calculated from the ratio between the first derivatives of pressure and time (dP/dt_max_), normalized by the pressure peak of the same test and expressed as percentage pressure fall per 10 ms^12^^,^ 13 (Figure 2). For subjects whose T_total_ in their best test was higher than 500 ms, their second or third highest SNIP values were used for quantification, whereas those who did not meet this criteria in any of their three best tests were excluded and statistics were recalculated for analysis. The Shapiro-Wilk test was used to verify the normality of variables. SNIP and MRR and their absolute and normalized values were compared using a paired Student's t-test. Statistical significance was set at p>0.05. Statistical analysis was performed using GraphPad Prism^(r)^, version 5.0 (GraphPad Software, San Diego, CA, USA).

## Results

A total of 22 individuals were recruited. Of these, one was excluded for exhibiting FVC and FEV_1_ less than 80% of the predicted value and another because the SNIP curves obtained after complete assessment were not suitable for graph analysis, since the tests did not produce gentle, descending curves and displayed a biphasic peak. Anthropometric and lung function data are shown in Table 1. Mean SNIP values, absolute and normalized MRR, and T_total_ are displayed in Table 2.

**Table 1.** Descriptive analysis of anthropometric measurements and spirometry.

	**Male (N=9)**	**Female (N=11)**	**N=20**
Age (years)	23.2 (2.8)	22.8 (3.1)	23.0 (2.9)
Weight (Kg)	82 (9.0)	60.4 (7.9)	70.2 (13.6)
Height (cm)	177.9 (4.2)	164.8 (5.9)	170.7 (8.4)
BMI (Kg/m^2)^	25.9 (2.5)	22.2 (2.1)	23.8 (3.0)
FVC (L) [%_predicted_]	5.3 (0.6) [96 (9.2)]	3.7 (0.5) [92.8 (9.7)]	4.4 (1.0) [94.2 (9.4)]
FEV_1_(L) [%_predicted_]	4.4 (0.4) [94.4 (7.4)]	3.2 (0.5) [96.5 (9.7)]	3.8 (0.7) [95.4 (8.6)]
FEV_1_/FVC	0.8 (0.04)	0.9 (0.1)	0.9 (0.1)
MIP (cmH_2_O)	156.4 (55.1)	109.1 (22.1)	131.0 (48.0)

Data are presented as mean and standard deviation. BMI: body mass index; FVC: forced vital capacity; %predicted: percentage of predicted value; FEV_1_: forced expiratory volume in one second; MIP: maximal inspiratory pressure.

**Table 2.** Sniff nasal inspiratory pressure, maximum relaxation rate, and sniff total duration time.

	**Without DiaphC**	**With DiaphC**	**p**	**Mean Difference**	**95% CI**
SNIP (cmH_2_O)	-100 (27.1)	-72.8 (22.3) *	< 0.0001	-27.15	-32.84 -21.46
MRR (cmH_2_O/s)	962.3 (326.5)	647.3 (218.6) *	< 0.0001	315.0	+195.5 +434.5
MRR normalized	-9.7 (2.6)	-8.9 (1.5)	0.19	-0.7750	-1.97 +0.42
T_TOTAL_ *sniff* < 500 ms	12 (60%)	20 (100%)	-		-
T_TOTAL_ *sniff* > 500 ms	8 (40%)	-	-		-

Data are presented as mean and standard deviation. SNIP: sniff nasal inspiratory pressure; MRR: maximum relaxation rate; diaphC: diaphragmatic activation control. *p<0.05.

A significant reduction was observed in SNIP values obtained for maneuvers performed using DiaphCtrl (p<0.0001) and in absolute MRR calculated from the same maneuvers (p<0.0001). There was no significant difference between MRRs when normalized (Table 2).

**Figure 3.**
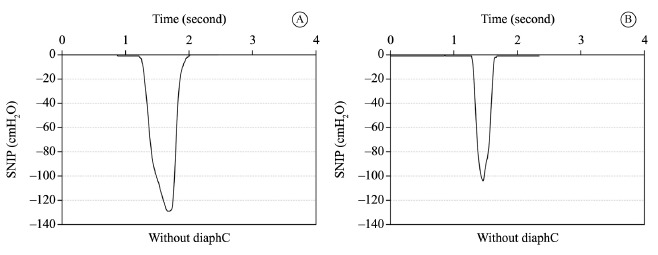
Graphic representation of Sniff Nasal-Inspiratory Pressure (SNIP) kinetics. Figure (A) without diaphragmatic control and (B) with diaphragmatic control in the same subject. On the left, there is a peak pressure of ~130 cmH_2_O and a total duration time of sniff ~630 milliseconds; on the right, there is a peak pressure of ~103 cmH_2_O and a total duration time of ~350 milliseconds. diaphC: diaphragmatic activation control.

Figure 3 illustrates the kinetic pattern of SNIP tests for a single individual with and without DiaphCtrl, showing the shape of the SNIP curve and T_total_ for the maneuver. Maneuvers with DiaphCtrl exhibited a lower T_total_ than those executed without DiaphCtrl. In maneuvers without DiaphCtrl, 40% (n=8) of subjects obtained a T_total_ higher than 500 ms. However, in maneuvers with DiaphCtrl (assessment B), 100% (n=20) of subjects achieved a T_total_ lower than 500 ms, as shown in Table 2.

## Discussion

The aim of this study was to assess the influence of diaphragmatic control (DiaphCtrl) on SNIP and MRR in healthy subjects. It was found that T_total_ was lower in tests with DiaphCtrl; the SNIP value was significantly lower compared to tests in which DiaphCtrl was not applied; and absolute MRR also declined, since it is directly proportional to the pressure obtained (MRR=dP/dt_max_). However, when normalized by the pressure peak of the same test, MRR exhibited similar behavior in all the situations proposed.

Based on these results, it can be hypothesized that DiaphCtrl enables subjects to perform a SNIP test exhibiting ballistic characteristics, as described in the literature. Moreover, despite producing lower SNIP values than tests without DiaphCtrl, all tests with diaphragmatic control met the requirements previously described to be deemed technically acceptable.

Regarding the higher values measured in tests executed without DiaphCtrl, a significant proportion (40%) were not considered technically acceptable, since the T_total_ obtained was greater than 500 ms. In maneuvers without instruction on DiaphCtrl, longer test times may be due to greater recruitment of (accessory) inspiratory muscles in the rib cage, which is not considered suitable for the SNIP test. The same logic can be applied to the decline in SNIP value. In other words, since diaphragmatic control results in lower recruitment of accessory muscles, there are fewer muscle fibers generating force during the maneuver and the peak value is therefore lower, but still reflects that the technique was executed correctly.

The relationship between the force generated by the diaphragm and other respiratory muscles, as well as the pressure obtained, may vary depending on the maneuver executed as a result of the chest wall movements prompted by specific respiratory muscle recruitment patterns. As such, it is suggested that the different SNIP values observed in the two assessments (A and B) of this study may be due to lower recruitment of the accessory muscles of respiration.

Previous studies using electromyography^10^ have shown that the diaphragm is the most active muscle during the sniff maneuver, which also recruits the scalene^23^, sternomastoid^10^^,^ 23, and intercostal muscles^9^^,^ 10. Nava et al.^10^ assessed three different breathing muscle recruitment maneuvers and demonstrated that sniffing exhibits greater diaphragm activation. The authors^10^ also suggest that the inspiratory muscle recruitment pattern in the sniff and Müller's maneuver are similar, but differ in terms of diaphragm activation. Katagiri et al.^23^ studied the activation of accessory muscles during sniffs and found that the scalene muscles were active during low- and high-intensity sniffs, whereas the sternomastoid muscle was only recruited during high-intensity sniffs (≥40 cmH_2_O).

Based on observation of the sniff maneuver performed by the individuals in this study, we found that muscles other than the respiratory muscles were recruited when insufficient instructions were given on diaphragm control, including the use of paraspinal muscles noted in a brief chest extension performed by the subject when executing the sniff.

The visible recruitment of accessory muscles in assessment A decreased after subjects were trained in diaphragmatic breathing (DiaphCtrl), evaluated by abdominal movement paired with sniff execution. Thus, when the maneuver was performed with DiaphCtrl, the SNIP value declines, likely due to reduced activity by other breathing muscles and the recruitment of more fibers, characterizing isometric muscle contraction.

Despite this qualitative observation, it is important to note that there are no clear reports on executing the technique. According to American Thoracic Society/European Respiratory Society (ATS/ERS)^16^ guidelines, subjects are asked to perform a maximal sniff followed by a slow, sustained expiration. It is also suggested that the sniff maneuver requires little instruction and practice and was performed this way in assessment A. Although the SNIP technique is entirely noninvasive, previous studies have shown a strong relationship between nasal airway and esophageal pressure, measured during sniffs by a balloon catheter in healthy subjects (r=0.99, p<0.001) and those with neuromuscular dysfunction (r=0.96, p<0.001)^24^. Given this behavior, the SNIP test is widely used in clinical practice, largely because it is easily executed by children and patients with neuromuscular diseases^3^^,^ 5 ^,^ 25.

As previously mentioned, SNIP values were considerably higher during the test performed without DiaphCtrl than those recorded after instruction on diaphragmatic breathing. However, a significant proportion of individuals in the DiaphCtrl test exceeded the time of 500 ms recommended in the literature^11^. Without instruction on applying diaphragmatic contraction during the SNIP tests, 40% of the subjects assessed were excluded from the study. The subtle change in execution provided by diaphragmatic control reduced T_total_ in 100% of subjects, and all tests complied with the acceptability criteria of duration of up to 500 ms. Thus, reducing test times by applying DiaphCtrl during the sniff maneuver means more subjects meet the inclusion criteria for data quantification described in previous studies^12^^,^ 13 ^,^ 26.

Kyroussis et al.^11^^,^ 12 and García-Rio et al.^13^ found that the MRR of respiratory muscles measured by the SNIP is highly correlated (healthy subjects: r=0.99, p<0.001; patients with neuromuscular diseases: r=0.98, p<0.001) with measurement of this rate by esophageal pressure and has been reported as a predictor of muscle fatigue. Thus, the decline in this rate represents muscle fatigue resulting from a reduction in force generation and/or increase in T_total_ for the test (MRR=dP/dt).

The MRR represents the kinetics involved in relaxation in the SNIP test. When normalized by the peak pressure obtained in the same maneuver, the rate behaved similarly in all the situations applied in this study, with and without DiaphCtrl. In other words, instruction on executing diaphragmatic control during the sniff did not change the kinetics of relaxation, but increased the number of technically acceptable tests. This is because, despite the significant decrease in test time with DiaphCtrl, the peak pressure obtained also declined, meaning the relationship between these two measurements (normalized MRR) remained relatively unchanged in static terms. Therefore, it is important to highlight that, notwithstanding the similarity between the normalized MRRs obtained with and without DiaphCtrl, 40% of the tests would not be acceptable for quantification according to current criteria. In order to remove any doubt regarding changes to test kinetics, the second or third best test for each individual whose T_total_ exceeded 500 ms was selected in an attempt to include them according to criteria. Nevertheless, 10% of subjects were still above the cutoff time applied. As such, these were excluded and all data reanalyzed, with no significant changes in the results, i.e. the normalized MRR of respiratory muscles remained unchanged.

The study exhibited limitations in its assessment format. Since subjects were assessed in a single session, the protocol adopted involved executing 5 SNIP maneuvers with and without DiaphCtrl, despite previous studies suggesting a minimum of 10 maneuvers to achieve the maximum SNIP value^27^. Lofaso et al.^27^ observed an intrasession learning effect in the sniff maneuver after the tenth repetition (between the 10^th^ and 20^th^ repetitions) when several maneuvers are repeated in the same session. In the present study, there was a subtle difference in execution of the sniff maneuver between assessments A and B. Thus, we opted for only 5 repetitions to avoid, as much as possible, the likelihood of an intrasession learning effect. However, it is important to note that the same conditions were applied in both assessments to enable comparison. Additionally, as a result of the study design adopted, it is impossible to prove that some of the subjects assessed did not voluntarily apply diaphragmatic breathing before being instructed to do so. However, a post-hoc analysis demonstrated that a significant proportion of the sample did not spontaneously use visual diaphragm contraction. As such, we feel that simply applying this technique can improve the results of the maneuver.

## Conclusions

Encouraging diaphragm contraction during the SNIP test influences the inspiratory pressure obtained, which is lower when diaphragmatic breathing is applied, but does not affect normalized MRR. As such, diaphragmatic control should be used, since it ensures that the values obtained in testing conform to the guidelines described in the literature.
