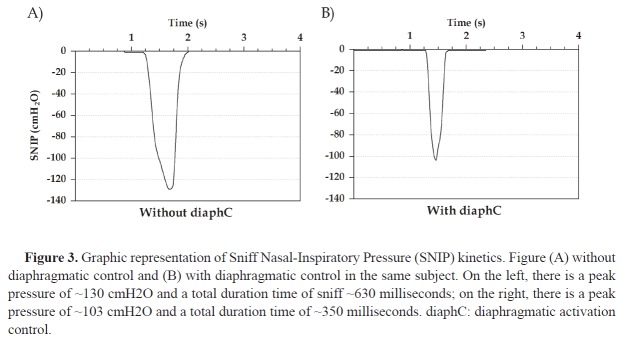# Erratum

**DOI:** 10.1590/bjpt-rbf.2014.0101er

**Published:** 2016

In the article **Effects of diaphragmatic control on the assessment of sniff nasal inspiratory pressure and maximum relaxation rate**, DOI: http://dx.doi.org/10.1590/bjpt-rbf.2014.0101, published in the Brazilian Journal of Physical Therapy, Volume 20, Number 1, page 96-103, on page 98 and 100, it reads:

**Figure:**
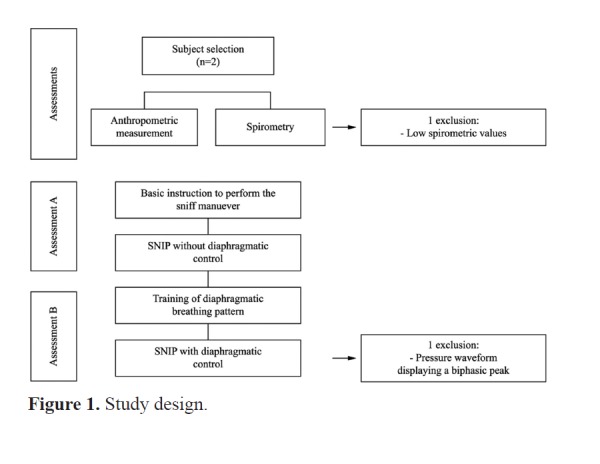

**Figure:**
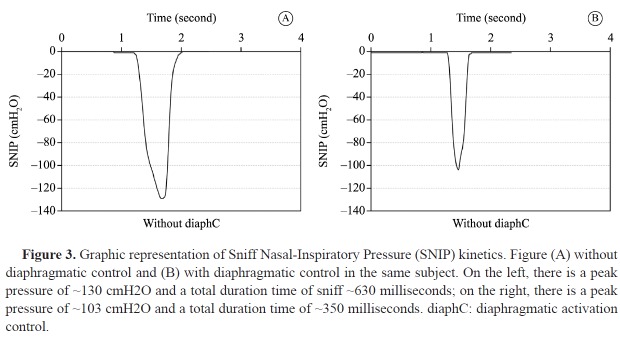

It should read:

**Figure:**
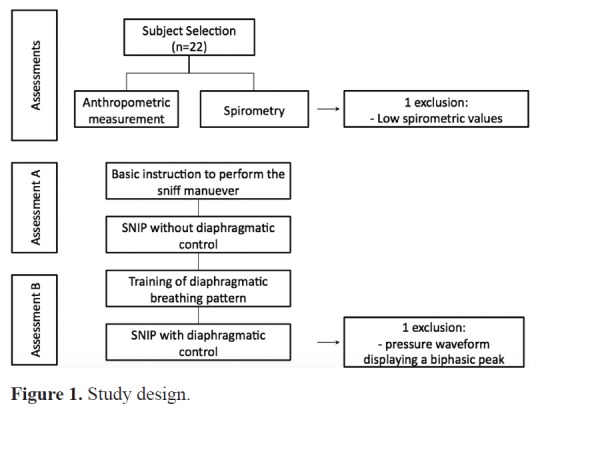

**Figure:**